# The expression of YWHAZ and NDRG1 predicts aggressive outcome in human prostate cancer

**DOI:** 10.1038/s42003-020-01645-2

**Published:** 2021-01-22

**Authors:** Sofia Lage-Vickers, Juan Bizzotto, Maria Pia Valacco, Pablo Sanchis, Sergio Nemirovsky, Estefania Labanca, Carlos Scorticati, Osvaldo Mazza, Antonina Mitrofanova, Nora Navone, Elba Vazquez, Javier Cotignola, Geraldine Gueron

**Affiliations:** 1 Laboratorio de Inflamación y Cáncer, Departamento de Química Biológica, Facultad de Ciencias Exactas y Naturales, Universidad de Buenos Aires, Buenos Aires, C1428EGA Argentina; 2 Instituto de Química Biológica de la Facultad de Ciencias Exactas y Naturales (IQUIBICEN), CONICET-Universidad de Buenos Aires, Buenos Aires, C1428EGA Argentina; 3 Department of Genitourinary Medical Oncology and The David H. Koch Center for Applied Research of Genitourinary Cancers, The University of Texas MD Anderson Cancer Center, Houston, TX 77030 USA; 4 Cátedra de Urología, Hospital de Clínicas, Buenos Aires, C1120AAR Argentina; 5 Department of Biomedical and Health Informatics, Rutgers School of Health Professions, Rutgers Cancer Institute of New Jersey, New Jersey, NJ 07101 USA

**Keywords:** Molecular biology, Cancer

## Abstract

Some prostate cancers (PCas) are histo-pathologically grouped within the same Gleason Grade (GG), but can differ significantly in outcome. Herein, we aimed at identifying molecular biomarkers that could improve risk prediction in PCa. LC ESI–MS/MS was performed on human PCa and benign prostatic hyperplasia (BPH) tissues and peptide data was integrated with *omic* analyses. We identified high *YWHAZ* and *NDRG1* expression to be associated with poor PCa prognosis considering all Gleason scores (GS). *YWHAZ* and *NDRG1* defined two subpopulations of PCa patients with high and intermediate risk of death. Multivariable analyses confirmed their independence from GS. ROC analysis unveiled that *YWHAZ* outperformed GS beyond 60 months post-diagnosis. The genomic analysis of PCa patients with *YWHAZ* amplification, or increased mRNA or protein levels, revealed significant alterations in key DNA repair genes. We hereby state the relevance of *YWHAZ* in PCa, showcasing its role as an independent strong predictor of aggressiveness.

## Abstract (web summary)

Using integrative tissue-omics, Lage-Vickers, Bizzotto et al. classify prostate cancer patients into high *YWHAZ* and *NDRG1* expression subgroups that correlate with a high and intermediate risk of disease progression. This study identifies molecular biomarkers associated with aggressive prostate cancer.

## Introduction

When prostate cancer (PCa) is diagnosed at a local or regional stage, the 5-year survival rate approaches 100%. But PCa is often symptomless in its early stages and once it has metastasized, survival rates decrease to 30%^1^. Hence, screening for aggressive cancer at early stages may be crucial to improve survival.

Widespread use of prostate-specific antigen (PSA) levels for screening has led to a large increase in the incidence of diagnosed PCa and a reduction in both, advanced disease and PCa mortality rates^2^. However, the overtreatment of PCa is widely recognized^3^. The difficulty in preventing overtreatment is the current inability to distinguish men who will have an indolent disease from those who will have aggressive disease. For men with newly diagnosed PCa, the strongest predictor is the Gleason Grade (GG). GG histologically groups PCa and presupposes an outcome for each grade within certain margins. However, in many cases, the outcome does not conform to these expectations and is usually more compromised^4^. Because of tumor heterogeneity that is inadequately captured by the biopsies, between 25 and 50% of biopsies with certain GG come from men with higher-grade PCa^4–6^. This is known as “upgrading” between biopsy and prostatectomy, indicating that patients diagnosed with indolent PCa might have higher-grade and subsequently higher-risk cancers.

Hence, efficient PCa management should encompass not only early accurate diagnosis but also the identification of prognostic factors which help in foreseeing the outcome for all individual cases. Clinical, genomic, and/or radiological biomarkers are the key to appropriate risk stratification. Genomic biomarkers are being developed for screening for lethal disease subtypes, monitoring of PCa recurrence after initial treatments, prognosis, and prediction of drug efficacy^7^. The application of translational molecular profiling in PCa may, in the near future, have the potential to enhance clinical management. In this regard, the literature reflects some interesting avenues such as the prostate cancer antigen 3 (PCA3) score^8^, the Prostate Health Index (phi)^9^, and the Oncotype Dx^10^. While PCA3 has a better diagnostic performance than PSA^11–13^, it does not add predictive value for GG or tumor stage^14,15^. In direct comparisons, the phi is a better predictor of PCa at initial biopsy, and therefore more suitable for screening^16–18^. Oncotype Dx for Prostate is used to further stratify low to low-intermediate risk PCa by calculating a Genomic Prostate Score. However, to prove clinical utility, potentially novel prognostic molecular markers will need to provide added, independent value, in multivariable analysis, beyond PSA, pathologic stage, and GG.

In this work, we undertook an in-depth mass spectrometry approach to profile proteomes from formalin-fixed paraffin-embedded (FFPE) specimens of prostate adenocarcinomas and benign prostate hyperplasias (BPHs), with available disease stage, Gleason score (GS), and patient age. The objective of our study was to identify novel biomarkers for risk stratification of PCa with an eye toward those that could behave independently from GS and further recognize intermediate GSs that may be more likely to progress. These molecular biomarkers might improve the prediction of lethal disease and provide insight into the biological mechanisms underlying the strong relation of GS and disease progression.

## Results

### Proteomic analysis of FFPE PCa and BPH

**Fig. 1:**
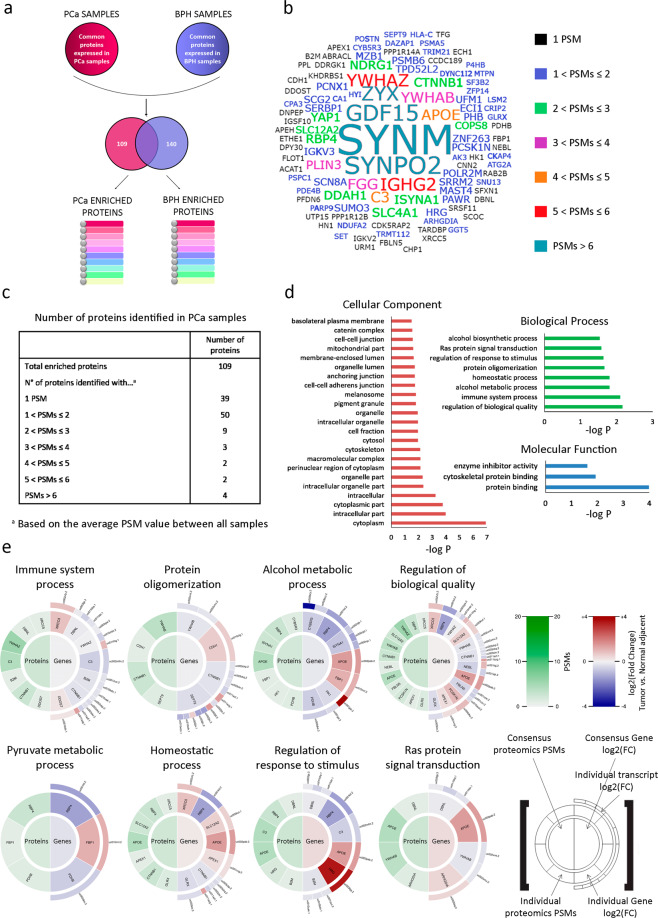
Identification of PCa enriched proteins. **a** Data analysis was based on label-free spectral counting, obtaining an average of 540 and 536 proteins per sample in PCa and BPH tissue proteomes, respectively. Two proteomic data sets were generated (PCa and BPH), taking into account the proteins that were found in at least 50% of the samples of each type of tissue, and that were also not shared between both groups. 109 proteins were enriched in PCa samples, while 140 proteins were enriched in BPH samples. **b** Tag cloud network of the 109 PCa enriched proteins identified consistently across the proteomics analyses performed in PCa compared to BPH FFPE human tissue samples. The font size increases proportionally to the average PSMs of these proteins across all the PCa samples. The color reflects the same information. **c** Semi-quantitative analysis of proteins identified by LC ESI–MS/MS classified by the number of PSMs obtained from PCa tissue samples. Results represent the average PSMs for each group. **d** GO analysis of PCa enriched proteins using the DAVID software which includes only significant categories (−log *P* ≥ 1.5) from cellular components, biological process, and molecular functions. **e** Protein levels (PSMs) and gene differential expression (PCa vs. normal adjacent tissue, *TCGA-PRAD*) of PCa enriched proteins were grouped by GO terms from the biological process category using DAVID software and visualized in proteogram plots. These graphs are circular heat-plots depicting both protein PSMs on the left semicircle and gene expression fold change (as log_2_ (fold change)) on the right semicircle, with the center indicating the averages. For the gene expression, a half outer semicircle was added to include each gene’s transcript’s expression level. PSM data were locally generated while gene expression data was gathered from *TCGA-PRAD*.

To identify potential PCa biomarkers for risk stratification, we performed LC ESI–MS/MS in human PCa (*n* = 10) and BPH (*n* = 10) archival tissues. The proteomics yield averaged 540 and 536 proteins per sample in PCa and BPH tissues, respectively. To analyze these proteins, we proceeded as described in the data analysis pipeline (Supplementary Fig. 1a). We then selected proteins enriched in PCa samples compared with BPH samples (PCa enriched protein data set, *n* = 109) (Fig. 1a). We subjected the candidate proteins to clinical validation in extended cohorts of PCa patients (32 data sets; 5974 samples) (Supplementary Fig. 1b).

### An integrated proteomics and transcriptomic atlas of PCa

To assess the clinical significance of the PCa enriched protein data set, we first generated a tag cloud network of the 109 proteins (Fig. 1b). These data are summarized in Fig. 1c, and complete lists of enriched proteins identified within each data set are presented in Supplementary Data 1 and 2.

Gene ontology (GO) classifications for the top biological processes (BP) categories of the PCa enriched protein list included RAS protein signal transduction, regulation of biological processes, and homeostasis (Fig. 1d). We then examined simultaneously the PCa proteome within the enriched GO categories, layering it with transcriptomic data from the *TCGA-PRAD* data set (*n* = 499)^19^. The patterns in the proteograms highlighted some accordance between proteins from the PCa enriched protein data set compared with the corresponding gene expression analysis from the *TCGA-PRAD* data set (Fig. 1e).

### Analysis of the PCa proteome through multiple microarray data sets and selection of candidate biomarkers

**Fig. 2:**
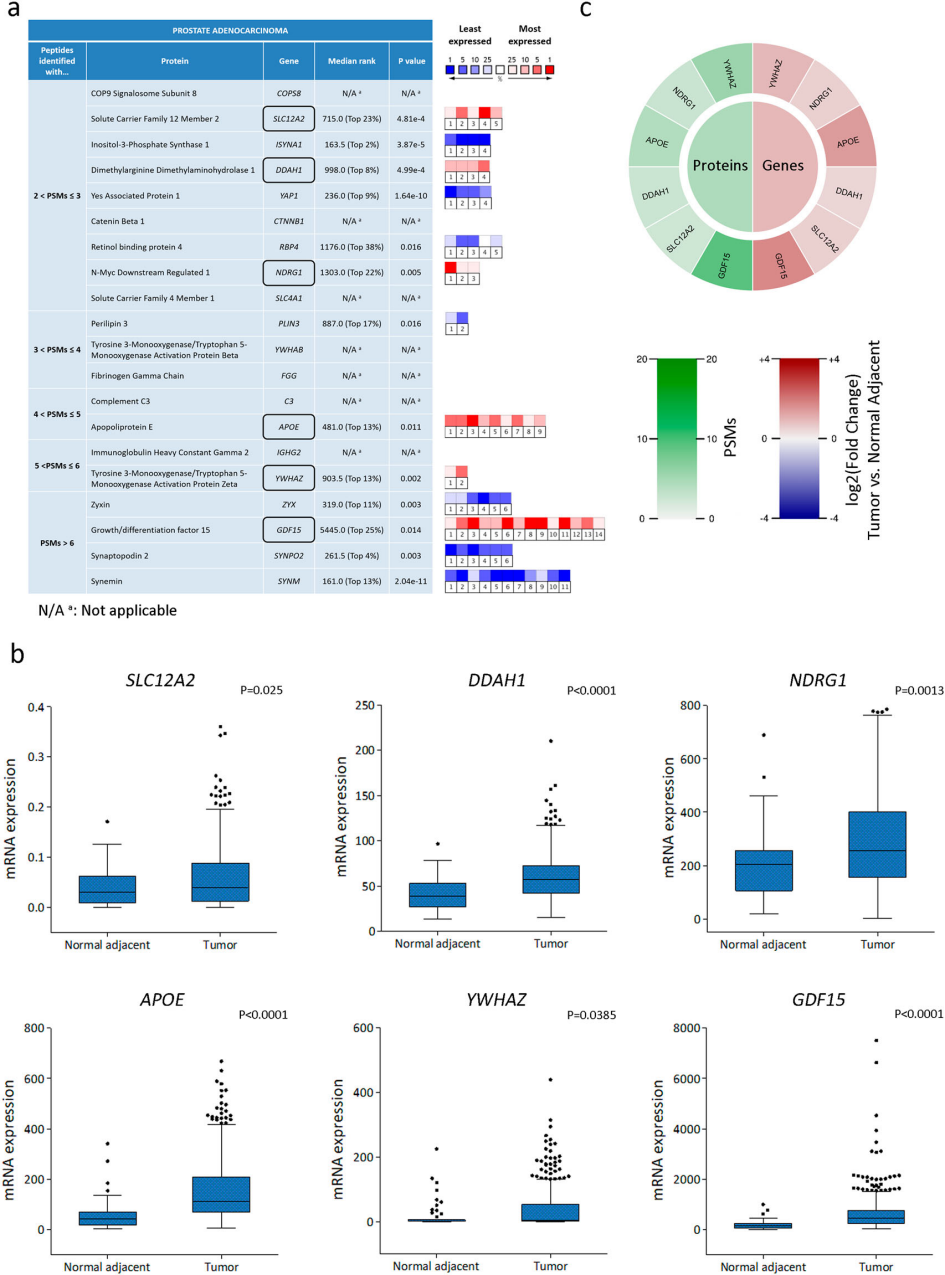
Correlation between proteomics and trancriptomics data for the PCa selected enriched proteins. **a** Summary table showing the gene symbol & name, median gene rank and corresponding *P*-value obtained with *Oncomine* (*n* = 1128) for the PCa enriched proteins identified with a higher number of PSMs. The median rank for a gene is the rank for the gene across each of the analyses for the 16 data sets assessed. The *P*-value for a gene is the *P*-value for the median ranked analysis. The heatmap on the right indicates the level of expression for each gene in each study selected (blue: least expressed, red: most expressed). Each square represents each *Oncomine* study that met our eligibility criteria and thresholds (see “Methods” section). Colors are *z*-score normalized to depict relative values within a row. Proteins found in PCa samples and upregulated in prostate carcinoma vs. normal prostate gland (*Oncomine*) are boxed in black. N/A ^a^: not applicable. **b** Tukey box plots showing median gene expression of *SLC12A2*, *DDAH1*, *NDRG1*, *APOE*, *YWHAZ*, and *GDF15* comparing tumor vs. Normal adjacent tissues using the *TCGA-PRAD* data set (*n* = 499). The top and bottom of each rectangular box represent the 75th and 25th percentiles, respectively, with the median indicated with a solid line inside the box. Horizontal bars extending from each box represent more extreme values defined as 1.5-times the interquartile range (25th percentile subtracted from the 75th percentile) above the 75th percentile or below 25th percentile. Circles represent outliers. Student’s *t*-test was used to ascertain statistical significance. Statistical significance was set at *P* ≤ 0.05. **c** Protein levels (PSMs) and gene differential expression (PCa vs. normal adjacent tissue, *TCGA-PRAD*) of PCa selected enriched proteins were put together in a proteogram plot.

In order to prioritize candidate biomarkers for PCa, we selected proteins within the PCa proteomes identified with more than 2 Peptide Spectral Matches (PSMs) (Fig. 1c). This filter resulted in the selection of 20 proteins (Fig. 2a). We next subjected our candidate list to bioinformatics analysis using *Oncomine*^20^. We selected 16 data sets (*n* = 1128) that analyze gene expression between PCa and normal prostate gland samples (Supplementary Table 1). The expression profile for most of these genes in the PCa enriched list identified with more than 2 PSMs showed significant dysregulation in PCa compared with normal gland (Fig. 2a). However, only *SLC12A2* (*P* = 4.81e−4), *DDAH1* (*P* = 4.99e−4), *NDRG1* (*P* = 0.005), *APOE* (*P* = 0.011), *YWHAZ* (*P* = 0.002), and *GDF15* (*P* = 0.014) displayed significant upregulated gene expression levels when comparing PCa vs. normal prostate gland (Fig. 2a, black boxes). The meta-analysis combining data from the independent data sets showed that the above-mentioned dysregulated genes lie within the 25% of the most consistently highly expressed genes across this comparison. Of note, *DDAH1*, *APOE*, and *YWHAZ* lie within the top 13% (Fig. 2a).

Further, in-depth analysis of *TCGA-PRAD* data revealed significantly higher gene expression profiles for *SLC12A2* (*P* = 0.025), *DDAH1* (*P* < 0.0001), *NDRG1* (*P* = 0.0013), *APOE* (*P* < 0.0001), *YWHAZ* (*P* = 0.0385), and *GDF15* (*P* < 0.0001), in PCa tumor tissue compared with non-tumoral adjacent tissue (Fig. 2b). Hence, the pattern evident in the proteogram, highlights a concordance between proteins enriched in PCa and the corresponding gene expression levels from the *TCGA-PRAD* data set (Fig. 2c).

### Identification of risk predictors of PCa

**Fig. 3:**
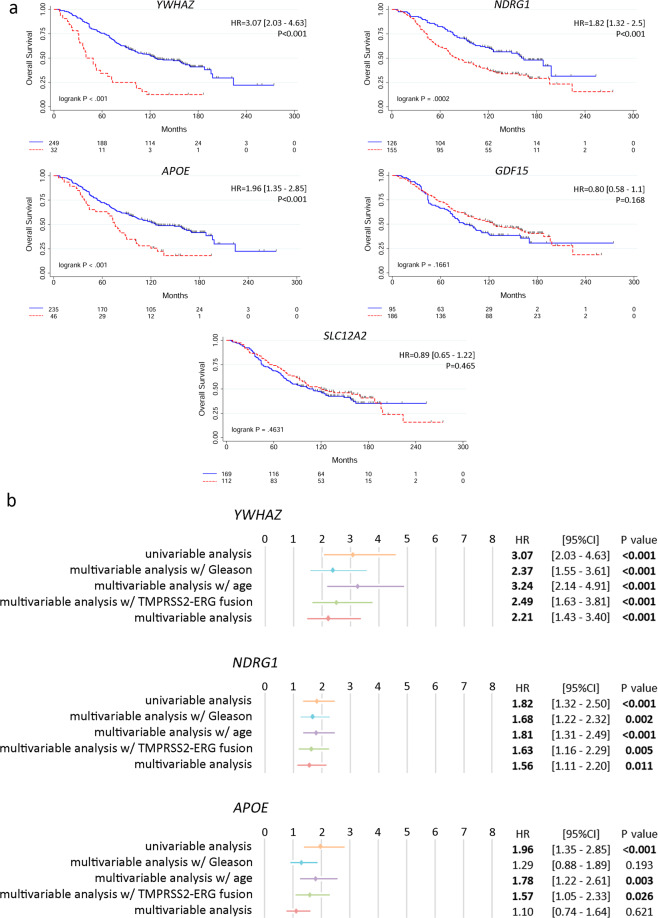
Overall survival (OS) of PCa patients naive of treatment based on *YWHAZ*, *NDRG1*, *APOE*, *GDF15*, and *SLAC12A2* expression (*Sboner* data set, GSE16560, *n* **=** 281). **a** Kaplan–Meier curves for OS for PCa patients segregated based on the gene expression levels for *YWHAZ*, *NDRG1*, *APOE*, *GDF15* and *SLAC12A2*. OS of patients with high (red dotted-lines) vs. low (blue full-lines) expression for each gene. **b** Multivariable analyses presented by forest plots including each gene with GS, age or *TMPRSS2-ERG* fusion or all the variables together. Statistical significant associations are bolded. Multivariable analysis w/Gleason (light blue) = adjusted for GS (6; 7 (3 + 4); 7 (4 + 3); 8–10). Multivariable analysis w/age (purple) = adjusted for age at diagnosis (age groups: 50–70; 70–80; 90–100). Multivariable analysis w/*TMPRSS2-ERG* fusion (green) = adjusted for *TMPRSS2-ERG* fusion. Multivariable analysis (red) = adjusted for GS, age at diagnosis and *TMPRSS2-ERG* fusion. GS = Gleason score. HR = hazard ratios [95% confidence interval] for the univariable analysis. All comparisons consider low expression patients as the reference group. *P* = Cox proportional hazard model *P*-value. Statistical significance was set at *P* ≤ 0.05.

We next evaluated the overall survival (OS) in PCa patients that had undergone TURP or adenoma enucleation with a high or low expression for each gene. The *Sboner* data set^21^ demonstrated that higher expression of *YWHAZ*, *NDRG1*, and *APOE* is significantly associated with poor OS in PCa (*P* < 0.001 for all genes) (Fig. 3a). Interestingly, *YWHAZ* is an androgen-responsive gene that activates proliferation, cell survival, and androgen receptor transcriptional activity^22^, *NDRG1* is a downstream target of *c-MYC* proto-oncogene^23^, and *APOE* is associated with fat metabolism and cancer^24^. *DDAH1* was not available in this data set (Fig. 3a).

To validate the potential of these biological markers to improve risk stratification in PCa, multivariable analyses were performed in the presence of clinico-pathological parameters previously associated with increased death risk. These parameters included GS, age group, and *TMPRSS2-ERG* fusion (Fig. 3b). High *YWHAZ* and *NDRG1* expression significantly correlated with poor prognosis. Both genes behaved independently from the patient’s GS, age, or *TMPRSS2-ERG* fusion (Fig. 3b). When we further adjusted the model to include simultaneously all variables, the associations remained significant for both genes (*P* < 0.001 and *P* = 0.011, respectively) (Fig. 3b). Although high *APOE* expression was associated with poor OS and the multivariable analyses including either age or *TMPRSS2-ERG* fusion were significant, no independence from the clinico-pathological parameters was observed when considering all variables simultaneously (Fig. 3b). These results may suggest that dysregulation of *APOE* may be either accompanying GS or maybe just a molecular/metabolic consequence of it.

**Fig. 4:**
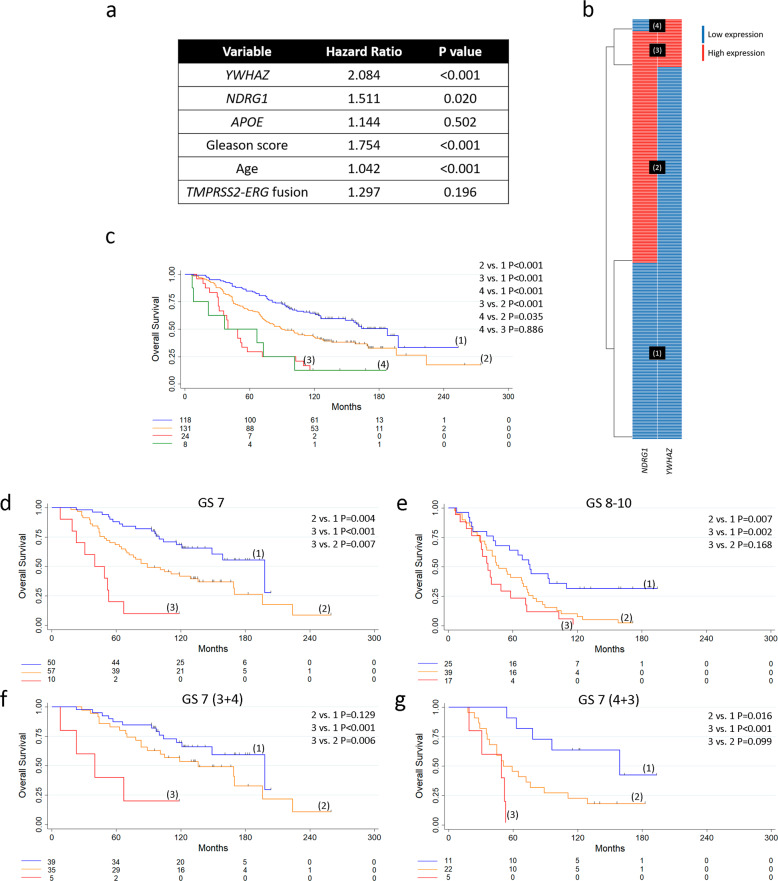
*YWHAZ* and *NDRG1* as risk stratification genes in PCa patients naive of treatment (*Sboner* data set, GSE16560, *n* **=** 281). **a** Multivariable analyses based on gene expression of *YWHAZ*, *NDRG1*, *APOE*, GS, age and *TMPRSS2-ERG* fusion for patients with PCa. *P* = Cox proportional hazard model *P*-value. **b** Heatmap depicting low (blue) or high (red) *NDRG1* and *YWHAZ* gene expression for patients with PCa. Patient subgroups are presented in black boxes. **c**–**g** OS of patients with low *YWHAZ* and *NDRG1* gene expression (1), high *NDRG1* gene expression (2), high *YWHAZ* gene expression (4), and high *YWHAZ* and *NDRG1* gene expression (3). Kaplan–Meier curves for OS for PCa patients segregated based on gene expression levels for *YWHAZ* and *NDRG1* (**c**), gene expression levels for *YWHAZ* and *NDRG1* for GS 7 (**d**) and GS 8–10 (**e**), and gene expression levels for *YWHAZ* and *NDRG1* for GS 7 (3 + 4) (**f**) and GS 7 (4 + 3) (**g**). GS = Gleason score. All comparisons consider low expression patients as the reference group. *P* = pairwise log-rank *P-*values. Statistical significance was set at *P* ≤ 0.05.

Further, we considered a multivariable Cox proportional hazard model including *YWHAZ*, *NDRG1*, *APOE*, GS, age, and *TMPRSS2-ERG* fusion in treatment-naive PCa. Results showed that only *YWHAZ*, *NDRG1*, GS, and age could be independent predictors of death risk (*P* < 0.001, *P* = 0.02, *P* < 0.001, and *P* < 0.001, respectively) (Fig. 4a). Hence, we dropped *APOE* and categorized PCa patients based on *YWHAZ* and *NDRG1* gene expression levels. The heatmap depicts patient subgroups with (1) low *YWHAZ* and *NDRG1* expression (*n* = 118); (2) high *NDRG1* and low *YWHAZ* expression (*n* = 131); (3) high *NDRG1* and *YWHAZ* expression (*n* = 24), and (4) low *NDRG1* and high *YWHAZ* expression (*n* = 8) (Fig. 4b). Next, we performed OS analyses of these patient subgroups. Patients in groups 2, 3, and 4 had significantly decreased OS compared with patients in group 1 (*P* < 0.001 for all comparisons) (Fig. 4c). No significance was observed when comparing OS between patients in groups 3 and 4 (Fig. 4c). These results evidenced three clear and distinct subpopulations of PCa patients with low (group 1), intermediate (group 2), and high (groups 3 and 4) risk of death. Since groups 3 and 4 presented a similar OS, we inferred that *NDRG1* has no significant effect on patients’ OS with high *YWHAZ* expression. Hence, *YWHAZ* seemed to be the main driver for the increased risk of death in patients with both high *NDRG1* and *YWHAZ*. In light of these results, we merged these two subgroups of patients for further analysis and named it group 3. Next, when categorizing patients based on GS, we were able to see once more how these subgroups separated into low, intermediate, and high-risk patients (Fig. 4d, e). In particular, for GS 7, we could observe high statistical significance for all comparisons (2 vs. 1 *P* = 0.004; 3 vs. 1 *P* < 0.001; 3 vs. 2 *P* = 0.007), reinforcing the power of these two biomarkers to define three subgroups of PCa risk (Fig. 4d). We further split the GS 7 patients into GS 7 (3 + 4) and GS 7 (4 + 3). KM curves evidenced the power of *YWHAZ* to predict a worse outcome for both GS: 7 (3 + 4) (3 vs. 1 *P* < 0.001) (Fig. 4f) and 7 (4 + 3) (3 vs. 1 *P* < 0.001) (Fig. 4g). *NDRG1* only defined an intermediate risk group for 7 (4 + 3) (2 vs. 1 *P* = 0.016) (Fig. 4g).

When performing the same analysis stratifying patients based on age, high *YWHAZ* expression was also associated with significantly poorer outcomes (3 vs. 1 *P* < 0.001 for both age groups) (Supplementary Fig. 2).

**Fig. 5:**
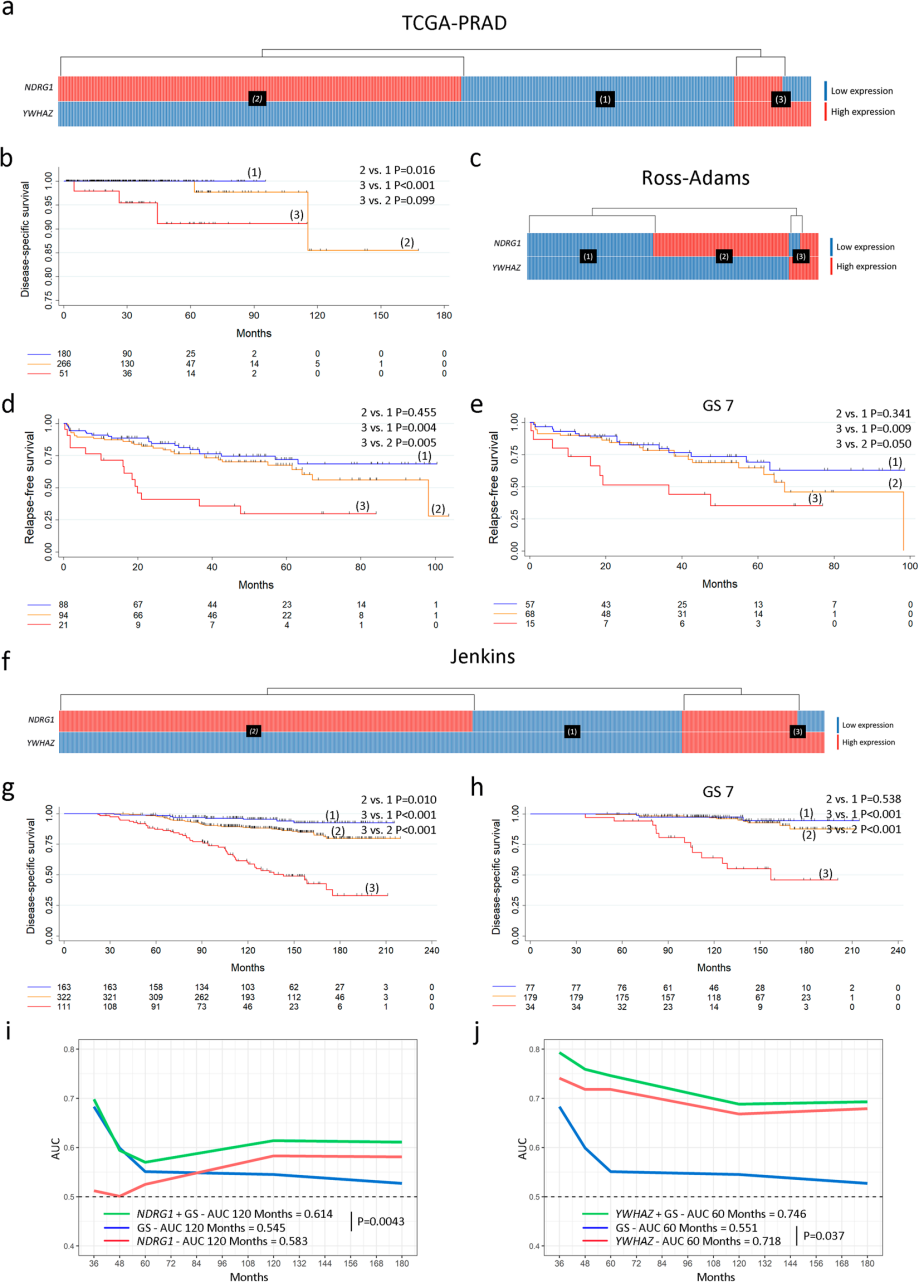
Validation of *YWHAZ* and *NDRG1* as risk stratification genes in PCa patients (*TCGA-PRAD*, *n* **=** 499; *Ross-Adams*, GSE70770, *n* **=** 206; and *Jenkins*, GSE10645, *n* **=** 596). **a**, **c**, **f** Heatmaps depicting low (blue) or high (red) *NDRG1* and *YWHAZ* gene expression for patients with PCa according to the *TCGA-PRAD* (**a**), *Ross-Adams* (**c**), and *Jenkins* (**f**) data sets. Patient subgroups are presented in black boxes: low *YWHAZ* and *NDRG1* expression (1), high *NDRG1* expression (2), and high *YWHAZ* expression (3). **b**, **d**, **g** Kaplan–Meier curves for DSS (*TCGA-PRAD*) (**b**), RFS (*Ross-Adams*) (**d**), and DSS (*Jenkins*) (**g**), for PCa patients segregated based on gene expression levels for *YWHAZ* and *NDRG1*. **e**, **h** Kaplan–Meier curves for RFS (*Ross-Adams*) (**e**) and DSS (*Jenkins*) (**h**), for PCa patients segregated based on gene expression levels for *YWHAZ* and *NDRG1* in GS 7. *P* = pairwise log-rank *P*-values. **i**, **j** Time-dependent AUC curves measured from 36 to 180 months reflecting the performance of GS (blue), *NDRG1* (**i**) or *YWHAZ* (**j**) (red), and *NDRG1* + GS (**i**) or *YWHAZ* + GS (**j**) (green) in PCa patients. AUC = area under the ROC curve. *P* = *P*-value for DeLong’s test for two ROC curves. GS = Gleason score. All comparisons consider low expression patients as the reference group. Statistical significance was set at *P* ≤ 0.05.

Since two independent PCa prognostic factors were delineated using the *Sboner* data set as our training data set, we sought to predict survival based on *YWHAZ* and *NDRG1* in three other independent non-overlapping validation data sets: *TCGA-PRAD* (*n* = 499), *Ross-Adams* (*n* = 206)^25^, and *Jenkins* (*n* = 596)^26^. The heatmaps depict patient subgroups with (1) low *YWHAZ* and *NDRG1* expression; (2) high *NDRG1* and low *YWHAZ* expression; (3) high *YWHAZ* expression regardless of *NDRG1* expression (Fig. 5a (*TCGA-PRAD*), c (*Ross-Adams*), f (*Jenkins*)). Similar results were obtained in which *NDRG1* defined a PCa subgroup with intermediate-risk and *YWHAZ* defined a PCa subgroup with high risk, regarding disease-specific survival (DSS) (*TCGA-PRAD* and *Jenkins*) and relapse-free survival (RFS) (*Ross-Adams*) for all PCa patients (Fig. 5b, d, g). When subcategorizing by GS 7, only patients with high *YWHAZ* were associated with decreased RFS (*Ross-Adams*) (3 vs. 1 *P* = 0.009) and DSS (*Jenkins*) (3 vs. 1 *P* < 0.001) (Fig. 5e, h).

Further, we used the time-dependent univariable AUC metric to evaluate *YWHAZ* and *NDRG1* performance as predictors of death in PCa patients, using the *Jenkins* data set which contains DSS. Time-dependent AUC curves showed that, although *NDRG1* alone did not outperform GS, the model including both, *NDRG1* and GS, improved the prediction of DSS compared with GS alone beyond 120 months post-diagnosis (AUC = 0.614 for *NDRG1* + GS and AUC = 0.545 for GS at 120 months; *P* = 0.0043) (Fig. 5i). In the case of *YWHAZ*, the univariable AUC model comparing this factor with the univariable AUC for GS revealed that *YWHAZ* outperformed GS prognosis beyond 60 months (AUC = 0.718 for *YWHAZ* and AUC = 0.551 for GS at 60 months; *P* = 0.037) (Fig. 5j). The model including both, GS and *YWHAZ*, did not present significant differences compared with the predictive value of *YWHAZ* alone (Fig. 5j). Thus, *YWHAZ* rises as a potential predictor of aggressiveness in PCa.

### Genomic landscape of PCa patients with *YWHAZ* alterations

We extended the bioinformatics analysis of *YWHAZ* using *cBioPortal for Cancer Genomics*^27^. 11 PCa data sets were selected that met our eligibility criteria (*n* = 2820) (Supplementary Table 2). The most frequent genetic alteration found was gene amplification (Supplementary Fig. 3a). Results showed significantly reduced disease-free survival in patients with *YWHAZ* gene alteration (*P* = 8.141e−3) (Supplementary Fig. 3b). The only data set available for direct correlation of exome data with RNAseq data was *TCGA-PRAD* (*n* = 499). There was a significant direct correlation between amplification for *YWHAZ* and mRNA levels (*P* < 0.001) (Supplementary Fig. 3c) and a positive correlation between 14-3-3ζ/δ levels and *YWHAZ* expression (Spearman coefficient = 0.29, *P* = 2.84e−8) (Supplementary Fig. 3d). Interestingly, the Reverse Phase Protein Array (RPPA) from *TCGA-PRAD* showed that patients with high 14-3-3ζ/δ levels had poorer disease-specific survival (DSS) (*P* = 0.021) (Supplementary Fig. 3e).

**Fig. 6:**
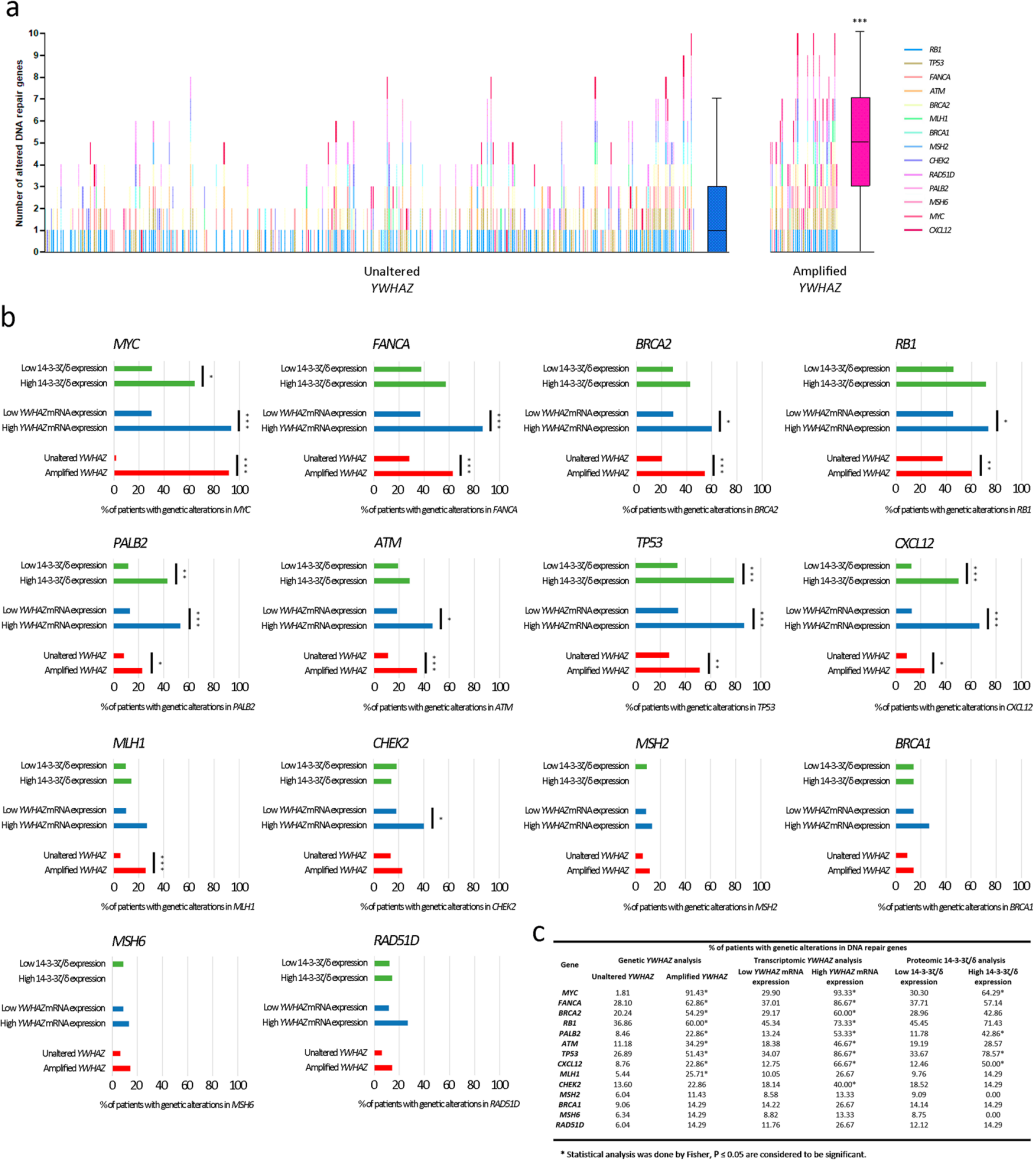
Genetic alterations (amplification, gain, shallow deletion, deep deletion, and point mutations) in DNA repair genes in PCa patients with *YWHAZ* amplifications and high YWHAZ mRNA and protein levels (*TCGA-PRAD* data set, *n* **=** 499). **a** Mutational landscape analysis (amplification, gain, shallow deletion, deep deletion, and point mutations) of DNA repair genes in patients with no alterations in *YWHAZ* (*n* = 331) and with amplifications in *YWHAZ* (*n* = 35). Each vertical line is a patient and the different colors represent alterations in a different gene, as specified in the references. The graph includes box plots showing the median number of altered DNA repair genes. The top and bottom of each rectangular box represent the 75th and 25th percentiles respectively, with the median indicated with a solid line inside the box. Horizontal bars extending from each box represent more extreme values defined as 1.5-times the interquartile range (25th percentile subtracted from the 75th percentile) above the 75th percentile or below 25th percentile. Student’s *t*-test was used to ascertain statistical significance. **b** Bar plots representing the percentage of PCa patients that present genetic alterations in each DNA repair gene based on whether they have amplifications in *YWHAZ* (red bars), high or low *YWHAZ* mRNA levels (blue bars), and high or low 14-3-3ζ/δ protein levels (green bars). **c** Comparative table of the percentage of patients that present genetic alterations in each DNA repair gene based on whether they have amplifications in *YWHAZ*, high or low *YWHAZ* mRNA levels, and high or low 14-3-3ζ/δ protein levels. Fisher’s exact test was used to test the statistical significance of contingency tables of genetic alterations. Statistical significance was set at *P* ≤ 0.05. *P ≤ 0.05, **P ≤ 0.01, ***P < 0.001.

Our results prompted the question as to whether PCa patients with *YWHAZ* genetic alterations, high *YWHAZ* expression or high 14-3-3ζ/δ levels, harbored a differential genomic landscape. We focused our work on key DNA repair genes that were previously described to have a high prevalence of mutations and copy number alterations in studies on localized and metastatic PCa^28,29^. When analyzing the genetic alterations of patients with localized PCa from the *TCGA-PRAD* data set with gene amplification for *YWHAZ*, there was a significantly higher number of altered genes per patient with *YWHAZ* amplification compared with those with unaltered *YWHAZ* (*P* < 0.001) (Fig. 6a and Supplementary Fig. 4). From these genes, nine (*MYC*, *FANCA*, *BRCA2*, *RB1*, *PALB2*, *ATM*, *TP53*, *CXCL12*, and *MLH1*) had significantly more genetic alterations when *YWHAZ* was amplified (Fig. 6b, red bars, c). Further, when analyzing patients with high vs. low YWHAZ mRNA and protein expression, four of those nine genes (*MYC, PALB2, TP53*, and *CXCL2*) also had increased significant genomic alterations (Fig. 6b, blue and green bars, respectively, c). Accordingly, similar results were obtained when performing the same comparison at the genomic level for *YWHAZ* in patients with metastatic PCa using the *SU2C/PCF 2019* data set (*n* = 444)^30^ (Supplementary Fig. 5). These results suggest a link between *YWHAZ* alterations and genomic instability and raises the question as to whether *YWHAZ* may also be a driver of progression.

## Discussion

In this work, we are reporting the relevance of *YWHAZ*/14-3-3ζ/δ as an independent strong predictor of death in PCa. Furthermore, we have discovered that this factor outperforms GS prediction of DSS beyond 5 years after initial diagnosis.

Briefly, in this study, we have: (1) applied high throughput proteomics methods to discover signature protein biomarkers that may be useful when analyzing PCa, and (2) identified and validated risk stratification markers within PCa patient data sets. We resumed our results in 20-candidate signature proteins enriched in PCa compared with BPH. Of those, 55% (11/20) correspond to a subset of proteins directly associated with PCa, and ~85% (17/20) are known cancer markers or have been previously associated with cancer in general^31–35^. These results clearly highlight the robustness of our working strategy and methodology. When analyzing these 20 candidates, the following gene transcripts appeared to be increased in PCa compared with the normal prostate gland: *SLC12A2*, *DDAH1*, *GDF15*, *APOE*, *NDRG1*, and *YWHAZ*. Although we did not validate the MS results on the protein level, there are previous reports that show increased protein expression of DDAH1, GDF15, APOE, NDRG1, and 14-3-3ζ/δ in PCa tissues compared with benign prostate tissues^22,36–39^.

In this work, we further merged our FFPE human PCa tissue proteomes data with large PCa transcriptome data sets, OS, RFS, and DSS data, and refined markers that could predict PCa survival. The univariable analyses performed on a well-characterized cohort (the Swedish Watchful Waiting Cohort after TURP or adenoma enucleation), revealed a positive association of increased expression of *APOE*, *YWHAZ*, and *NDRG1* with poor OS. When performing multivariable analyses, only *YWHAZ* and *NDRG1* showed independence from GS, age at diagnosis, and *TMPRSS2-ERG* fusion in PCa patients. These two genes were able to categorize PCa patients in low, intermediate, and high risk of death. Moreover, this pattern was also observed with statistical significance for GS 7. Further, we split the GS 7 patients in GS 7 (3 + 4) and GS 7 (4 + 3) in line with the current classification GG 2 and GG 3, and once more, the KM curves still evidenced the power of *YWHAZ* to predict a worse outcome for both groups.

Since TURP might trigger tumor cell dissemination^40–43^, we might speculate that this could be one of the reasons for the short OS in this cohort. For this reason, we furthered the analysis using the *Jenkins* data set that contains DSS. The ROC analysis showed that, although *NDRG1* did not outperform GS, the model including GS + *NDRG1* improved the predictive value of aggressive disease compared with GS alone beyond 120 months post-diagnosis. High *NDRG1* expression relates to increased cell differentiation signals in various cancer cell lines and the suppression of tumor metastasis^23^. In particular, its role in PCa is controversial since there are both, reports suggesting NDRG1 as a tumor suppressor^44^, and as an oncogene^39,45^.

In the case of *YWHAZ*, this factor outperformed GS beyond 60 months post-diagnosis. Interestingly, the ROC curves did not showcase improved predictive value when adding GS to the *YWHAZ* model, further ascertaining this factor as a potential prognostic tool in the clinic. 14-3-3ζ/δ is an adapter protein implicated in the regulation of a large spectrum of both general and specialized signaling pathways. 14-3-3ζ/δ belongs to the 14-3-3 family of proteins that mediate signal transduction by binding to phosphoserine-containing proteins and is encoded by the *YWHAZ* gene. Increased expression of *YWHAZ* relates to tumor cell proliferation and malignant outcome of gastric carcinoma^46^. In localized PCa, Ruenauver et al. associate *YWHAZ* with PCa^47^, however, these authors fail to demonstrate its relevance as a prognostic factor independent from the common PCa clinico-pathological parameters.

We hereby state the relevance of *YWHAZ*/14-3-3ζ/δ in PCa showcasing its role as an independent strong predictor of death that outperforms GS. With the identification of the mutational landscape of organ-confined and advanced-stage disease, a major contribution has been made to the development of molecular biomarker profiling in addition to serum PSA. It is convenient that clinico-pathological parameters, imaging, and molecular markers are integrated together to better predict tumor behavior. *YWHAZ*/14-3-3ζ/δ could be a promising tool when taking into consideration the difficulties that PCa presents at the time of decision making.

It is worth mentioning that when evaluating the association of *YWHAZ* with survival, its genetic alteration was significantly related to poor prognosis. These observations made us speculate as to whether patients with *YWHAZ* genetic alterations, high *YWHAZ* gene expression, or high 14-3-3ζ/δ protein levels, harbored a differential genomic profile compared with PCa patients with no *YWHAZ* alterations. We centered our attention on key DNA repair genes associated with localized and metastatic PCa^28,29^. Interestingly, PCa patients with *YWHAZ* amplification harbored significantly more genetic alterations. Of note, *MYC*, *PALB2*, *TP53*, and *CXCL2*, also had increased genetic alterations when comparing patients with high vs. low YWHAZ mRNA and protein expression. In the case of *MYC*, almost 92% of PCa patients with amplified *YWHAZ* presented *MYC* alterations as opposed to only 1.8% of patients with no *YWHAZ* alteration in *TCGA-PRAD*. Although co-occurrence of *MYC* and *YWHAZ* alterations could be explained by previously reported 8q gains associated with tumor progression and poor prognosis in PCa^29^, it is worth noticing that *YWHAZ* amplification, or increased YWHAZ mRNA and protein levels, significantly correlated with genetic alterations in other genes that are located at different chromosomes. These results reflect that a subpopulation of PCa patients with high *YWHAZ/*14-3-3ζ/δ shows greater number of genetic alterations in key DNA repair genes. Future work should address whether *YWHAZ* may also be a driver of progression.

We acknowledge the limitation of validating the initial proteomics screening in PCa and BPH samples with transcriptomics data. However, since we were processing archival FFPE samples, we had to prioritize tissue for LC ESI–MS/MS. Of note, the *Sboner* data set (GSE16560) included TURP and adenoma enucleation specimens, which might introduce short overall survival bias.

## Methods

### Experimental design

The study aimed to identify potential biomarkers for PCa risk stratification. An in-depth proteomics analysis (LC ESI–MS/MS) was done on human PCa and BPH tissues (previously confirmed by histological analyses performed by pathologists at the Hospital de Clinicas), since normal prostate gland samples were not available. Formalin-fixed and paraffin-embedded (FFPE) section tissues from 20 patients (ten radical prostatectomy specimens of treatment-naive PCa patients and ten BPH patients) were analyzed. Three FFPE sections per patient were used for protein extraction. Disease- and patient-associated data including pathologic and clinical stage and patient age were also obtained (Supplementary Table 3). Samples with less than 250 peptides were excluded from the subsequent analysis. Enriched PCa and BPH protein lists were formulated considering peptides that were found at least in ≥50% of tissue samples and were not common to both groups (PCa and BPH enriched protein data sets, respectively). Subsequently, extended cohorts of PCa patients (32 data sets; 5974 samples) were used for validation of proteins of interest.

### FFPE processing

PCa and BPH FFPE tissue sections mounted on microscope slides were processed as previously described in Wakabayashi et al.^48^. PCa and BPH FFPE tissue sections were deparaffinized and rehydrated by successive washes in 100% *n*-octane (1 × 1 h), 100% (2 × 6 min), 96% (2 × 6 min), 70% (2 × 6 min), 50% ethanol (2 × 6 min), and water (3 × 6 min). After air-drying, the tissue sections were percolated with CelLytic MT Mammalian Tissue Lysis/Extraction Reagent (Sigma) for 90 min and harvested with a scalpel blade. The collected tissues were incubated on a heating block at 99 °C for 60 min and then sonicated for five cycles of 30 s ON, 30 s OFF. The debris was pelleted by spinning at 1500 × *g* at 4 °C for 20 min. The recovered proteins in the supernatant were quantified with a Pierce BCA Protein Assay kit.

### LC ESI–MS/MS analysis

The digests were analyzed by nanoLC-MS/MS in a ThermoScientific Q-Exactive Mass Spectrometer coupled to a nanoHPLC EASY-nLC 1000 (ThermoScientific). For the LC ESI–MS/MS analysis, ~1 μg of peptides was loaded onto the column and eluted for 120 min using a reverse-phase column (C18, 2 μm, 100 A, 50 μm × 150 mm), Easy-Spray Column PepMap RSLC (P/N ES801)) suitable for separating protein complexes with a high degree of resolution. The flow rate used for the nano column was 300 nl min-1 and the solvent gradient range was 7% B (for 5 min) to 35% B in 120 min. Solvent A was 0.1% formic acid in water whereas B was 0.1% formic acid in acetonitrile. The injection volume was 2 μl. The MS equipment has a high collision dissociation cell (HCD) for fragmentation and an Orbitrap analyzer (Q-Exactive-ThermoScientific Germany). A voltage of 3.5 kV was used for Electro Spray Ionization (ThermoScientific, EASY-SPRAY).

XCalibur 3.0.63 (ThermoScientific) software was used for data acquisition with a configuration that allows peptide identification at the same time as their chromatographic separation. A Data dependant method was used: Full-scan mass spectra were acquired in the Orbitrap analyzer. The scanned mass range was 400–1800 *m*/*z*, at a resolution of 70000 at 400 *m*/*z* and the twelve most intense ions in each cycle were sequentially isolated, fragmented by HCD, and measured in the Orbitrap analyzer. Peptides with a charge of +1 or with an unassigned charge state were excluded from fragmentation for MS2.

### Analysis of LC ESI–MS/MS data

Raw data generated with Xcalibur software was processed and analyzed with Proteome discoverer 2.1.1.21 with SEQUEST Search engine. Spectrum Selector node with default parameter settings was used to generate peak lists. Minimum and maximum precursor masses were set at 350 and 5000 with an S/N of 1.5. Data were searched against Uniprot Homo sapiens UP000005640 database, October 2017, with trypsin specificity (full cleavage) and a maximum of two missed cleavages per peptide. Carbamidomethylation of cysteine residues was set as a fixed modification and oxidation of methionine was set as variable modification. Proteome Discoverer searches were performed with a precursor mass tolerance of 10 ppm and product ion tolerance to 0.05 Da. Proteome Discoverer default settings were used: Target FDR = 0.01; *Z* = 1 High confidence XCorr 1.5; *Z* = 2 High confidence XCorr 2; *Z* = 3 High confidence XCorr 2.5; *z* ≥ 4 High confidence XCorr 3. Protein hits were filtered for high confidence peptide matches with a maximum protein and peptide false discovery rate of 1% calculated by employing a reverse database strategy.

### Proteograms

Proteograms were constructed as an adaptation from the web-based analytic tool Metabologram^49^. Protein levels (Peptide Spectrum Matches, PSMs) and differential gene expression (PCa vs. normal adjacent tissue, *TCGA-PRAD*)^19^ of PCa enriched proteins were grouped by GO Terms from the biological process category and visualized in proteogram plots. These are circular heat-plots depicting both protein PSMs on the left semicircle and gene expression fold change (as log_2_ (fold change)) on the right semicircle, with the center indicating the averages. For the gene expression, a half outer semicircle was added to include the expression level for each gene transcript. PSM data was locally generated while gene expression data was gathered from the publicly available *TCGA-PRAD* data set.

### Gene ontology (GO) analysis

GO analysis was performed using the Database for Annotation, Visualization, and Integrated Discovery (DAVID) v6.7. (Leidos Biomedical Research, Inc., Bethesda, MD, USA)^50^.

### Bioinformatics analysis

#### Information source and eligibility criteria (The Cancer Genome Atlas (TCGA))

We used the data set from the Prostate Adenocarcinoma Project of The Cancer Genome Atlas (*TCGA-PRAD*)^19^ that has gene expression data from 499 prostate tumor and normal adjacent tissue samples (last access: December 2019), measured by massively parallel sequencing (lluminaHiSeq). A descriptive table regarding patient characteristics at baseline (start of the follow-up for analyses) is depicted in Supplementary Table 4.

#### Information source and eligibility criteria (*Oncomine*) (*n* = 1128)

We searched the public cancer microarray database *Oncomine*^20^ (715 data sets and 68 tumor types; last access: December 2019) to identify expression microarray data sets that compared the expression of prostate adenocarcinoma vs. normal prostate gland. To be included in our study, a data set was required to (1) be generated from human prostate tumors, and (2) compare prostate adenocarcinoma vs. normal prostate gland. Differential genes were considered when: (1) they presented a *P*-value < 0.05 and (2) had an increase or decrease in expression ≥1.5 times and/or had a gene rank within the top 10%. Although the *P*-value criteria was strict for the data set selection, some genes were considered even if the fold change or the gene rank was <1.5% or >10%, respectively, when the gene showed a significant over or under expression. Genes were ranked by their *P*-value for every analysis scoring a gene rank. Median rank is the median *P*-value rank across data sets, for each gene assessed.

Search criteria: We performed a search for each gene using its HGNC gene symbol as the search term. The resulting studies were analyzed on the basis of healthy prostate gland vs. prostate adenocarcinoma. Cited literature was reviewed to confirm that the analysis was as documented in the *Oncomine* database.

#### Information source and eligibility criteria (GEO: Gene Expression Omnibus)

Gene expression data sets for primary PCa samples: To study the impact of the expression of the selected genes on the survival of patients, three data sets were selected according to the following criteria: (1) the study includes metadata for each patient, with ≥5 years of follow-up survival, (2) the study consists of ≥200 samples, and (3) the study is published and available on GEO (Gene Expression Omnibus).

Search/study selection:

- *Sboner 2010* (GSE16560)^21^: a PCa patient’s cohort that had undergone transurethral resection of prostate (TURP) or adenoma enucleation taken at the time of the initial diagnosis. It is comprised of 281 tumor tissue samples from men with PCa from the Swedish Watchful Waiting Cohort, with up to 30 years of clinical follow-up, with complete Illumina GPL5474 Human 6k Transcriptionally Informative Gene Panel data. A descriptive table regarding patient characteristics at baseline (start of the follow-up for survival analyses) is depicted in Supplementary Table 5.
- *Ross-Adams 2015* (GSE70770) GPL10558 series^25^: a PCa patient’s cohort with 206 tumor tissue samples from men with PCa who had undergone radical prostatectomy and clinical follow-up of 9 years, including relapse information (biochemical relapse). Biochemical relapse was defined according to European Guidelines as a persistent rise of PSA above 0.2 ng/ml. Tumor sample expression of 31,000 transcripts was measured by 47,000 probes using the Illumina HumanHT-12 V4.0 platform. A descriptive table regarding patient characteristics at baseline (start of the follow-up for survival analyses) is depicted in Supplementary Table 6.
- *Jenkins* (GSE10645) GPL5873 DASL Custom Prostate Panel^26^: a PCa patient’s cohort with 596 tumor tissue samples from men with PCa undergoing radical prostatectomy, with PCa specific death information and a mean follow-up of 20 years. A descriptive table regarding patient characteristics at baseline (start of the follow-up for survival analyses) is depicted in Supplementary Table 7.

#### Information source and eligibility criteria (*cBioPortal*)

We searched the cBio Cancer Genomics Portal^27^, an open-source cancer genomics data platform created by Memorial Sloan-Kettering Cancer Center (MSKCC), to analyze the selected genes’ most common mutations, copy number alterations, and gene expression in PCa (11 data sets, *n* = 2820 samples, last access: December 2019). The criteria for inclusion of the data sets in our analysis were as follows: (1) type of cancer: prostate adenocarcinoma or metastasis, (2) the study must be published, and (3) the study must consist of a sample number >50.

#### Information source and eligibility criteria (SU2C/PCF Dream Team 2019 data set (SU2C)) ^30^

We used the data set from the SU2C-PCF Dream Team: Precision Therapy for Advanced Prostate Cancer that has whole-exome sequencing of 444 castrate-resistant prostate cancer tumor/normal pairs. A descriptive table regarding patient characteristics at baseline (start of the follow-up for analyses) is depicted in Supplementary Table 8.

### Time-dependent ROC curves

TimeROC^51^ package was used for the estimation of time-dependent receiver operating characteristic (ROC) curve and area under time-dependent ROC curve (AUC) in the presence of censored data. Confidence intervals (CI) were computed using the iid-representation tool within the package. The confidence level was set to 0.95.

### Statistics and reproducibility

We used the web-based bioinformatic tool Nexus^52^ for the analysis of differentially expressed genes based on the comparison of prostate tumor and normal adjacent tissue samples (*TCGA-PRAD*) (expressed as log_2_ fold change).

GraphPad Prism software (La Jolla, CA, USA) was used to calculate student’s t-test for testing differences in gene expression across tissue samples, and percentage of the genome altered in patients with high or low *YWHAZ* or 14-3-3ζ/δ expression. Statistical significance was set at *P* ≤ 0.05.

Stata software (StataCorp LLC, Texas, USA) was used to explore the patients’ survival and to generate Kaplan–Meier curves. To find the cutoff value to stratify patients into two groups based on the expression levels of each gene, we used the Cutoff Finder tool^53^. For univariable and multivariable analyses of prognostic factors, log-rank test and Cox proportional hazard model regression were employed.

Fisher’s exact test was used to test the statistical significance of contingency tables of genetic alterations. Statistical significance was set at *P* ≤ 0.05.

One way ANOVA followed by a Tukey’s test was performed to assess significant differences when comparing gene expression, and percentage of the genome altered across samples with copy number alterations.

### Ethics statement

Written informed consent and institutional review board approval from the teaching hospital “Hospital de Clínicas José de San Martín” in Buenos Aires, Argentina, were acquired.

### Reporting summary

Further information on research design is available in the Nature Research Reporting Summary linked to this article.

## Supplementary information

Supplementary information

Description of Additional Supplementary Files

Supplementary Data 1

Supplementary Data 2

Supplementary Data 3

Reporting Summary

## Data Availability

The data sets generated during the current study are available in the ProteomeXchange (http://www.proteomexchange.org) repository, via the identifier PXD014291. Source data underlying plots shown in figures are provided in Supplementary Data 3. All other relevant data are available from the authors upon request.
