# Supplementary material for: The expression of YWHAZ and NDRG1 predicts aggressive outcome in human prostate cancer
Source: Commun Biol. 2021 Jan 22;4:103. doi: 10.1038/s42003-020-01645-2 (PMC7822895; doi:10.1038/s42003-020-01645-2)
Supplement: Supplementary file 1 — Supplementary information [file 42003_2020_1645_MOESM1_ESM.pdf]

The expression of YWHAZ and NDRG1 predicts aggressive outcome in human prostate cancer

*Sofia Lage-Vickers[1-2]; *Juan Bizzotto[1-2]; Maria Pia Valacco[1-2]; Pablo Sanchis[1-2]; Sergio Nemirovsky[1-2]; Estefania Labanca[3]; Carlos Scorticatti[4]; Osvaldo Mazza[4]; Antonina Mitrofanova[5], Nora Navone[3]; Elba Vazquez[1-2]; #Javier Cotignola[1-2]; #Geraldine Gueron[1-2]

[1]Universidad de Buenos Aires. Facultad de Ciencias Exactas y Naturales. Departamento de Química Biológica, Laboratorio de Inflamación y Cáncer, Buenos Aires C1428EGA, Argentina.

[2]CONICET-Universidad de Buenos Aires. Instituto de Química Biológica de la Facultad de Ciencias Exactas y Naturales (IQUIBICEN), Buenos Aires, C1428EGA, Argentina.

[3]Department of Genitourinary Medical Oncology and the David H. Koch Center for Applied Research of Genitourinary Cancers, The University of Texas MD Anderson Cancer Center, Houston, TX 77030, USA.

[4]Cátedra de Urología, Hospital de Clínicas, Buenos Aires C1120AAR, Argentina.

[5]Department of Biomedical and Health Informatics. Rutgers School of Health Professions. Rutgers Cancer Institute of New Jersey, NJ 07101, USA.

*Sofia Lage-Vickers and Juan Bizzotto contributed equally.

#Corresponding authors: Javier Cotignola and Geraldine Gueron.

Javier Cotignola and Geraldine Gueron jointly supervised this work.

Lead contact: Geraldine Gueron

e-mails: ggueron@gmail.com, jcotignola@qb.fcen.uba.ar

SUPPLEMENTARY TABLES AND FIGURES

Supplementary Tables

Supplementary Table 1. Expression microarray studies selected from the *Oncomine* platform comparing prostate adenocarcinoma *vs.* normal prostate.

Supplementary Table 2. Studies selected from the *cBioPortal for Cancer Genomics* platform summarizing the total number of cases with alterations for each study.

Supplementary Table 3. PCa FFPE tissue samples used for proteomic analysis.

Supplementary Table 4. *TCGA-PRAD* patients' characteristics at baseline.

Supplementary Table 5. *Sboner* patients' characteristics at baseline.

Supplementary Table 6. *Ross-Adams* patients' characteristics at baseline.

Supplementary Table 7. *Jenkins* patients' characteristics at baseline.

Supplementary Table 8. *SU2C/PCF* patients' characteristics at baseline.

Supplementary Figures

Supplementary Figure 1. Simplified schematic workflow of the protein extraction procedure.

Supplementary Figure 2. *YWHAZ* and *NDRG1* as risk stratification genes in PCa patients naïve of treatment (*Sboner* dataset, GSE16560, n=281).

Supplementary Figure 3. Exome, RNAseq and proteomic analyses for *YWHAZ* in PCa patients (*cBioPortal*).

Supplementary Figure 4. Genomic alterations (amplifications, gain, shallow deletions, deep deletions and point mutations) in PCa patients according to *YWHAZ* copy number, mRNA and protein expression (*TCGA-PRAD* dataset, n=499).

Supplementary Figure 5. Genomic alterations (amplifications, gain, shallow deletions, deep deletions and point mutations) in DNA repair genes in PCa patients with *YWHAZ* amplifications, or high *YWHAZ* mRNA levels, or high 14-3-3ζ/δ levels from the *SU2C/PCF* dataset (n=444).

N°	Dataset	Platform	Measured Genes	Reporter	N° Samples	GEO accession
1	Arreduani Prostate	HG U133 Plus 2.0 Array	19,574	54,675	21	GSE55945
2	Grasso Prostate	Agilent Human Genome 44K	19,189	41,000	122	GSE35988
3	Holzbeierlein Prostate	HG U95A-Av2 Array	8,603	12,651	54	N/A [a]
4	Lapointe Prostate	Undefined Platform	10,166	19,116	112	GSE3933
5	LaTulippe Prostate	HG U95A-Av2 Array	8,603	12,651	35	GSE68882
6	Liu Prostate	HG U133A Array	12,624	22,283	57	N/A [a]
7	Luo Prostate	Hu35k (A-D) and HG U95A-Av2 array	15,302	47,060	30	GSE68545
8	Magee Prostate	HumanGeneFL Array	5,338	7,133	15	N/A [a]
9	Singh Prostate	HG U95A-Av2 Array	8,603	12,651	102	GSE68907
10	Taylor Prostate	Undefined Platform	22,238	43,419	185	GSE21032
11	Tomlins Prostate	Undefined Platform	10,656	19,928	101	GSE6099
12	Vanaja Prostate	HG U133(A-B) Array	17,779	44,928	40	N/A [a]
13	Varambally Prostate	HG U133 Plus 2.0 Array	19,574	54,675	19	GSE3325
14	Wallace Prostate	HG U133 2.0 Array	12,603	22,283	89	GSE6956
15	Welsh Prostate	HG U95A-Av2 Array	8,603	12,651	34	N/A [a]
16	Yu Prostate	HG U95A-Av2 Array	8,603	12,651	112	GSE68555

Supplementary Table 1. Expression microarray studies selected from the *Oncomine* platform comparing prostate adenocarcinoma vs. normal prostate gland. N/A [a]: Not available

N°	Dataset	Study name	% Altered Cases	N° Samples
1	Prostate (MICH)	Metastatic Prostate Adenocarcinoma (MCTP, Nature 2012)	27%	61
2	Prostate (SU2C 2019)	Metastatic Prostate Cancer (SU2C/PCF Dream Team, PNAS 2019)	25%	444
3	Prostate (SU2C)	Metastatic Prostate Cancer (SU2C/PCF Dream Team, Cell 2015)	16%	150
4	Prostate (Broad/Cornell 2013)	Prostate Adenocarcinoma (Broad/Cornell, Cell 2013)	4%	57
5	Prostate (Broad/Cornell 2012)	Prostate Adenocarcinoma (Broad/Cornell, Nat Genet 2012)	0.9%	112
6	Prostate (FHCRC 2016)	Prostate Adenocarcinoma (Fred Hutchinson CRC, Nat Med 2016)	30%	141
7	Prostate (MSKCC 2010)	Prostate Adenocarcinoma (MSKCC, Cancer Cell 2010)	6%	174
8	Prostate (MSKCC 2014)	Prostate Adenocarcinoma (MSKCC, PNAS 2014)	1.9%	104
9	PRAD (MSKCC/DFCI 2018)	Prostate Adenocarcinoma (MSKCC/DFCI, Nature Genetics 2018)	6%	1,013
10	Prostate (Eur Urol 2017)	Prostate Adenocarcinoma (SMMU, Eur Urol 2017)	3%	65
11	Prostate (TCGA)	Prostate Adenocarcinoma (TCGA, Firehose Legacy)	8%	499

Supplementary Table 2. Studies selected from the *cBioPortal* for Cancer Genomics platform summarizing the total number of cases with alterations for each study.

PCa Sample N°	Stage	Gleason	Gleason	Age
33B_430	pT1a	5+3	8	92
34B_432	pT2aNx	2+3	5	74
36B_704	pT2aN0	3+3	6	58
37B_347	pT2aN0	3+2	5	54
34B_667	pT3Nx	3+4	7	67
40B_071	pT1b	3+4	7	65
40B_834	pT2aNx	2+5	7	62
33B_385	pT3bNx	3+3	6	60
33B_722	pT2aNx	3+2	5	65
96B_235	pT2cN0	2+5	7	70

Supplementary Table 3. PCa FFPE tissue samples used for proteomic analysis. Table contains for each sample processed, disease- and patient-associated data including tumor pathologic and clinical stage and patient characteristics. (PCa: prostate cancer; FFPE: formalin-fixed paraffin embedded).

TCGA-PRAD patients' characteristics at baseline

	#	Freq. (%)
**Diagnosis Age (41-78, mean: 60.6 years)**		
< 50	35	7
50-59	190	38
60-69	238	47.6
70-79	37	7.4
>79	0	0
**Race Category**		
N.A.	344	68.8
white	147	29.4
black or african american	7	1.4
asian	2	0.4
**Tumor Stage**		
T2a	13	2.6
T2b	10	2
T2c	165	33
T3a	159	31.8
T3b	136	27.2
T4	10	2
N.A.	7	1.4
**Radical Prostatectomy Gleason Score**		
6	45	9
7 (3+4)	149	29.8
7 (4+3)	101	20.2
8	64	12.8
9	137	27.4
10	4	0.8

Supplementary Table 4. *TCGA-PRAD* patients' characteristics at baseline (start of the follow-up analyses). Table contains age, race category, tumor stage and radical prostatectomy Gleason score data at diagnosis.

Sboner patients' characteristics at baseline

	#	Freq. (%)
**Diagnosis Age (51-91, mean: 73.99 years)**		
< 50	0	0.00
50-59	10	3.56
60-69	62	22.06
70-79	141	50.18
>79	68	24.20
N.A.	105	37.37
**Gleason Score**		
6	83	29.54
7 (3+4)	79	28.11
7 (4+3)	38	13.52
8	27	9.61
9	49	17.44
10	5	1.78
**TMPRSS2 – ERG fusion**		
positive	42	14.95
negative	239	85.05

Supplementary Table 5. *Sboner* patients' characteristics at baseline (start of the follow-up survival analyses). Table contains age, Gleason score and TMPRSS2-ERG fusion data at diagnosis.

Ross-Adams patients' characteristics at baseline

	#	Freq. (%)
**Diagnosis Age (41-73, mean: 60.1 years)**		
< 50	6	2.91
50-59	37	17.96
60-69	61	29.61
70-79	7	3.40
>79	0	0.00
N.A.	105	50.97
**Clinical Stage**		
T1	102	49.51
T2	72	34.95
T3	25	12.14
N.A.	7	3.40
**Gleason Score**		
5	2	0.97
6	35	16.99
7 (3+4)	102	49.51
7 (4+3)	40	19.42
8	13	6.31
9	10	4.85
10	1	0.49
N.A.	3	1.46
**PSA at Diagnosis**		
< 4	11	5.34
4 - 10	134	65.05
> 10	60	29.13
N.A.	1	0.49

Supplementary Table 6. *Ross-Adams* patients' characteristics at baseline (start of the follow-up survival analyses). Table contains age, clinical stage, Gleason score and PSA at diagnosis data.

Jenkins patients' characteristics at baseline

	#	Freq. (%)
**Age at radical retropubic prostatectomy (47-73, mean: 65.4 years)**		
< 50	6	1.01
50-59	105	17.62
60-69	314	52.68
70-79	171	28.69
>79	0	0.00
**Gleason Score**		
5	4	0.67
6	79	13.26
7	290	48.66
8	69	11.58
9	145	24.33
10	9	1.51
**Tumor Stage**		
T2N0	254	42.62
T3aN0	140	23.49
T3bN0	125	20.97
TxN+	77	12.92

Supplementary Table 7. *Jenkins* patients' characteristics at baseline (start of the follow-up survival analyses). Table contains age at radical retropubic prostatectomy, Gleason score and tumor stage data at diagnosis.

SU2C/PCF patients' characteristics at baseline

	#	Freq. (%)
**Diagnosis Age (39-89, mean: 60.7 years)**		
< 50	30	6.76
50-59	131	29.50
60-69	177	39.86
70-79	33	7.43
>79	8	1.80
N.A.	50	11.26
**Radical Prostatectomy Gleason Score**		
6	29	6.53
7	107	24.10
8	69	15.54
9	128	28.83
10	24	5.41
N.A.	87	19.59

Supplementary Table 8. *SU2C/PCF* patients' characteristics at baseline (start of the follow-up analyses). Table contains age and radical prostatectomy Gleason score data at diagnosis.

a

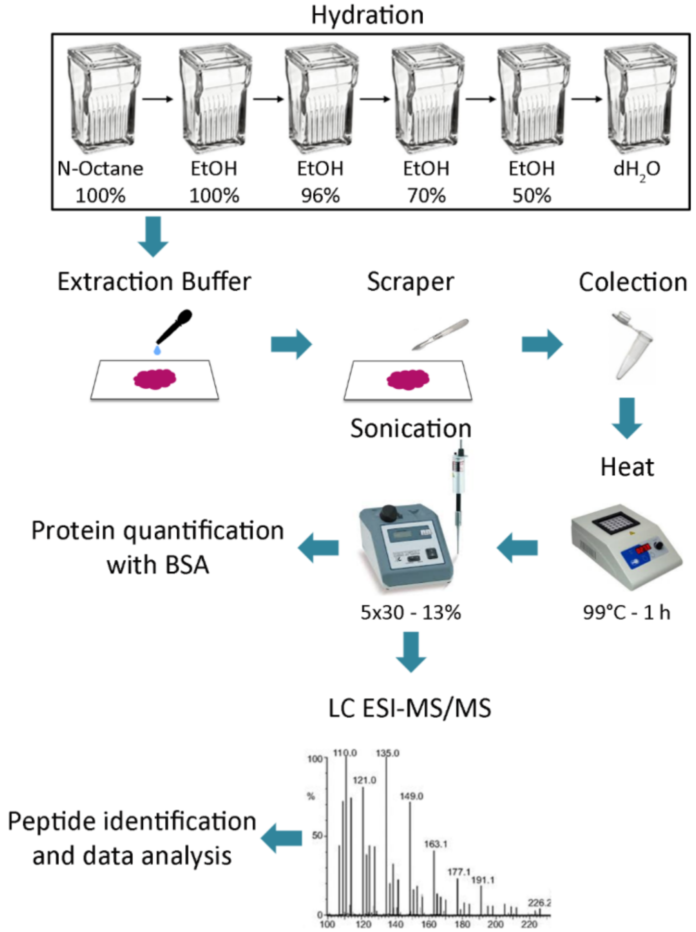

b

Oncomine
- 16 datasets. Microarrays.
- Prostate Adenocarcinoma vs. Prostate Gland.
- n=1,128

TCGA-PRAD
- 1 dataset. RNAseq.
- Tumor vs. Normal Adjacent samples.
- n=499

GEO
- Clinical metadata.
- *Sboner* (GSE16560; n=281)
- *Ross-Adams* (GSE70769; n=206)
- *Jenkins* (GSE10645; n=596)

cBioPortal
- 11 datasets. NGS.
- Tumor samples.
- n=2,820

SU2C/PCF
- 1 dataset. Whole exome sequencing.
- Castrate resistant prostate cancer tumor/normal pairs
- n=444

32 datasets - 5,974 samples

Supplementary Figure 1. Simplified schematic workflow of the protein extraction procedure. a) Samples were obtained from human PCa and benign prostatic hyperplasia (BPH) tissues using phase-transfer surfactant-aided extraction/tryptic digestion of formalin-fixed and paraffin-embedded sections mounted on microscope slides. The extraction procedure consisted of disruption of the crosslinked proteins which were then subjected to in depth proteomic analysis by electron spray ionization with tandem mass spectrometry (LC ESI-MS/MS). b) Summary of number of datasets, samples and sample type used in *Oncomine*, *TCGA-PRAD*, GEO, *cBioPortal* and *SU2C/PCF* used to validate the proteomics data.

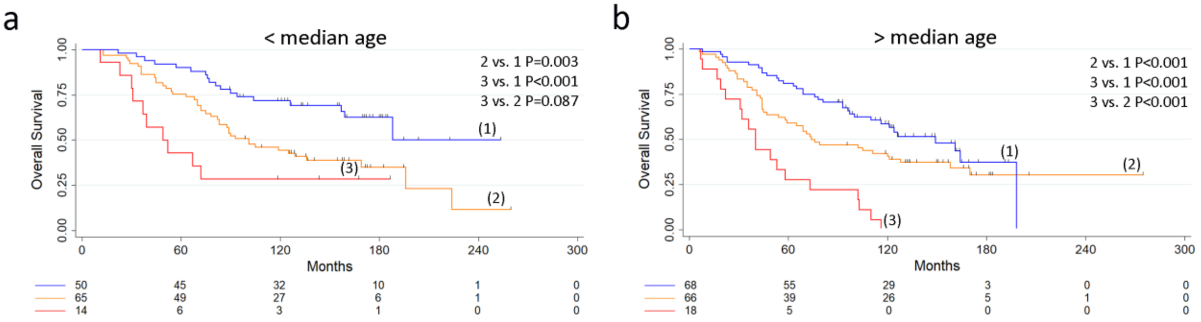

Supplementary Figure 2. *YWHAZ* and *NDRG1* as risk stratification genes in PCa patients naïve of treatment (*Sboner* dataset, GSE16560, n=281). Overall survival (OS) of patients with low *YWHAZ* and *NDRG1* mRNA expression (1), high *NDRG1* mRNA expression (2), and high *YWHAZ* mRNA expression (3) in the *Sboner* dataset. Kaplan–Meier curves for overall survival for PCa patients segregated based on gene expression levels for *YWHAZ* and *NDRG1* for patients < (a) and > (b) median age. Median age=74 years old. P = pairwise log rank P values. Statistical significance was set at P≤0.05.

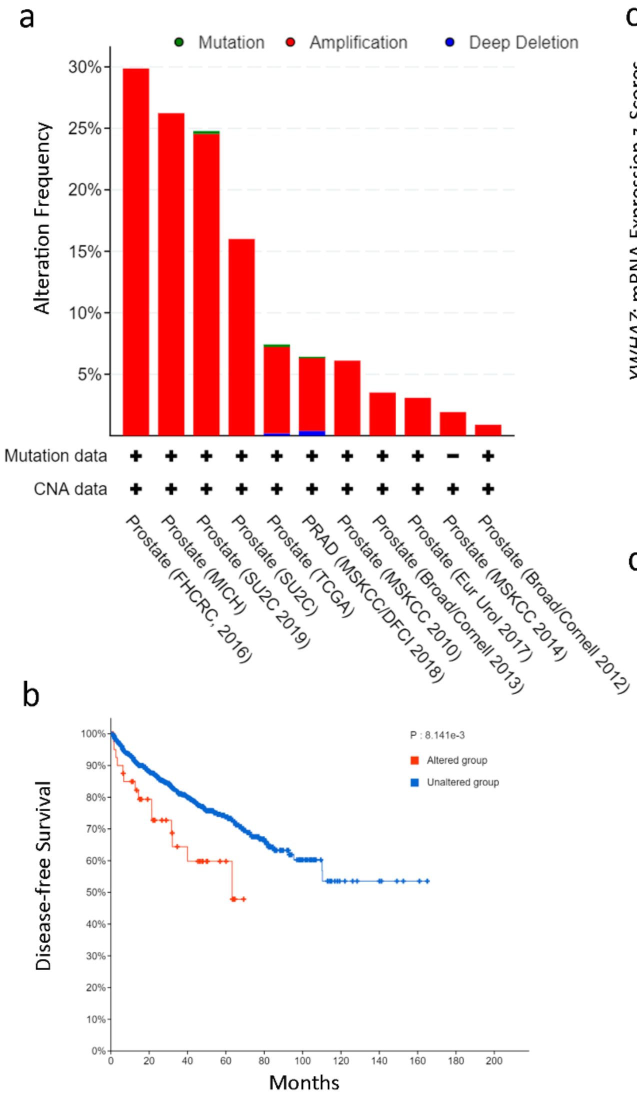

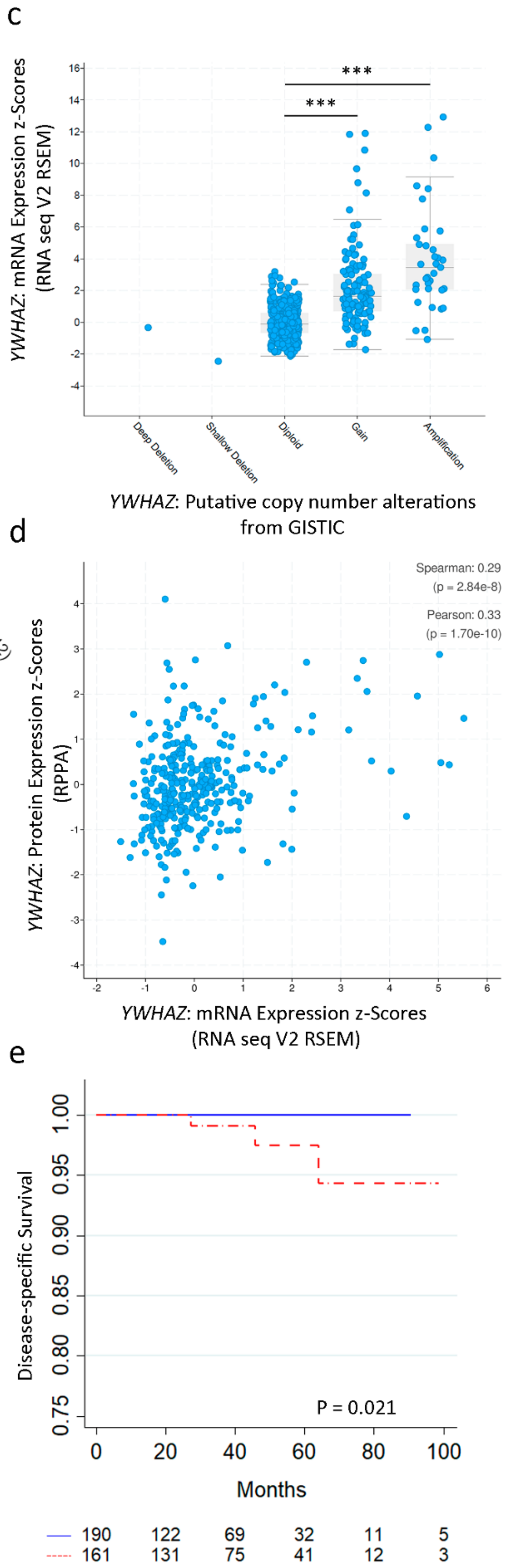

Supplementary Figure 3. Exome, RNAseq and proteomic analyses for *YWHAZ* in PCa patients (*cBioPortal*). a) The graph shows the percentage of altered cases (y axis) and the alteration type observed whether it corresponds to mutations (green), amplifications (red) or deep deletions (blue) for *YWHAZ* across the 11 datasets selected comparing prostate tumor tissue vs. normal prostate. b) Kaplan–Meier curve of disease-free survival for PCa patients segregated based on the presence (red line) or absence (blue line) of alterations in *YWHAZ* across the 11 selected datasets. c) Correlation between *YWHAZ* gene expression (y axis) and the alteration type whether it corresponds to deep deletions, shallow deletions, diploid, gain and amplifications (x axis) using the *TCGA-PRAD* dataset (n=499). One way ANOVA followed by a Tukey's test was performed to assess significant differences when comparing samples with copy number alterations to diploid samples. d) Spearman and Pearson correlation between 14-3-3ζ/δ protein and mRNA expression (*TCGA-RPPA*) (n=311). e) Kaplan–Meier curve of disease specific survival for PCa patients segregated based on high (red line) and low (blue line) 14-3-3ζ/δ protein expression according to the *TCGA-PRAD* dataset (n=499). P = pairwise log rank P values. Statistical significance was set at P ≤0.05. *** P≤0.001.

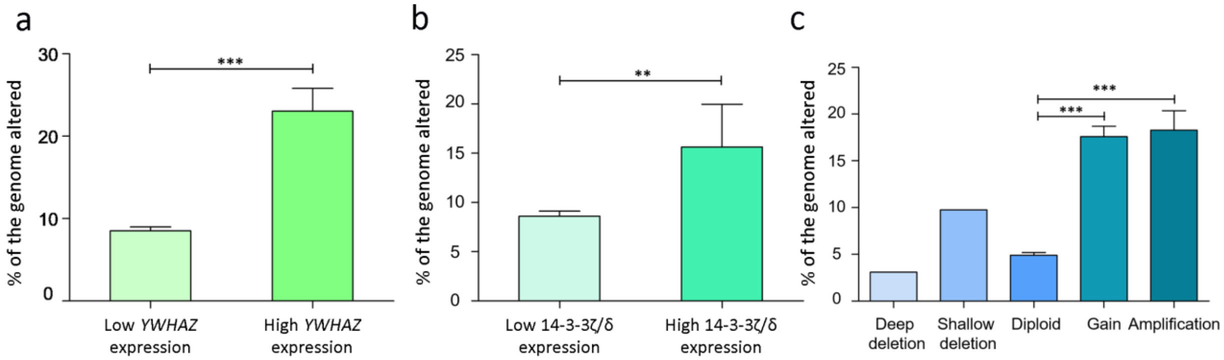

Supplementary Figure 4. Genomic alterations (amplifications, gain, shallow deletions, deep deletions and point mutations) in PCa patients according to *YWHAZ* copy number, mRNA and protein expression (*TCGA-PRAD* dataset, n=499). Percentage of the genome altered in PCa patients with a) high *vs.* low *YWHAZ* expression, b) high *vs.* low 14-3-3ζ/δ expression, and c) copy number alterations in *YWHAZ*. Results are shown as the mean ± s.e.m. Student's t-test was used to ascertain statistical significance. Statistical significance was set at P≤0.05. ** P≤0.01, *** P≤0.001.

a

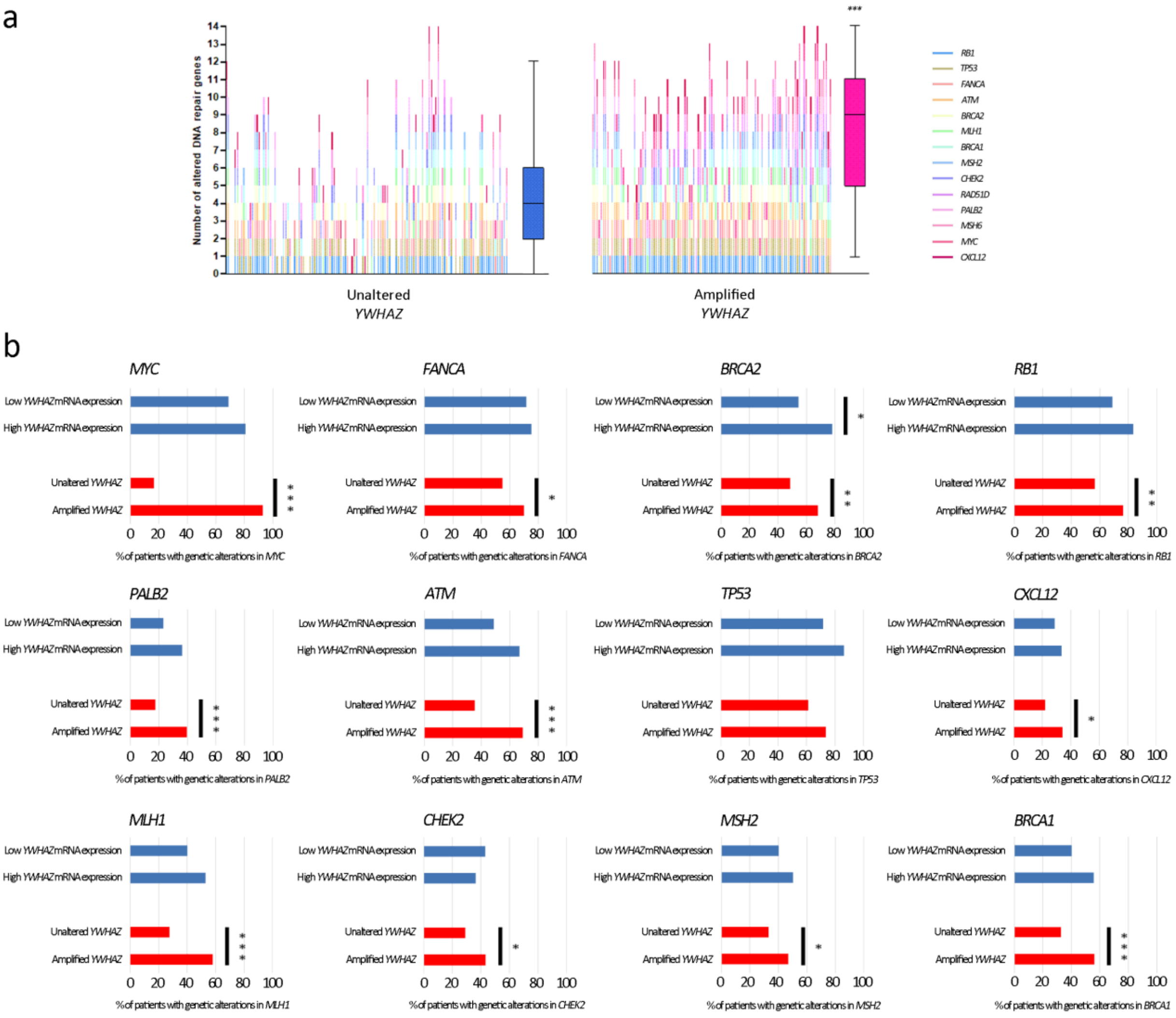

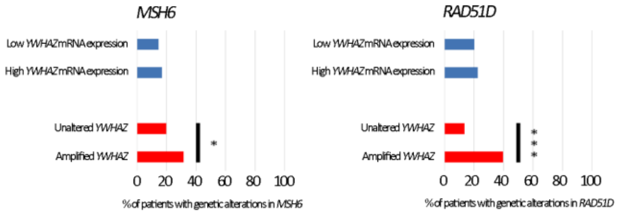

c

Gene	% of patients with genetic alterations			
	Genomic *YWHAZ* analysis		Transcriptomic *YWHAZ* analysis	
	Unaltered *YWHAZ*	Amplified *YWHAZ*	Low *YWHAZ* mRNA expression	High *YWHAZ* mRNA expression
MYC	21.87	92.66*	68.57	80.55
FANCA	54.69	69.72*	71.43	75.00
BRCA2	48.44	67.89*	54.28	77.78*
RB1	56.25	76.15*	68.57	83.33
PALB2	17.19	39.45*	22.85	36.11
ATM	25.16	68.81*	48.57	66.67
TP53	60.94	73.39	71.42	86.11
CXCL12	21.87	33.94*	28.57	33.33
MLH1	27.34	57.79*	40.00	52.78
CHEK2	28.91	43.12*	42.85	36.11
MSH2	32.81	46.79*	40.00	50.00
BRCA1	32.81	55.96*	40.00	55.55
MSH6	19.53	31.19*	14.28	16.67
RAD51D	13.28	39.45*	20.00	22.22

* Statistical analysis was done by Fisher, P ≤ 0.05 are considered to be significant.

Supplementary Figure 5. Genetic alterations (amplifications, gain, shallow deletions, deep deletions and point mutations) in DNA repair genes in PCa patients with *YWHAZ* amplifications, or high *YWHAZ* mRNA, or high 14-3-3ζ/δ levels from the *SU2C/PCF* dataset (n=444). a) Mutational landscape analysis (amplification, gain, shallow deletion, deep deletion and point mutations) of DNA repair genes in patients with no alterations in *YWHAZ* (n=128) and with amplification in *YWHAZ* (n=109). Each vertical line is a patient and the different colors represent alterations in a different gene, as specified in the references. The graph includes box plots showing the median number of altered DNA repair genes. The top and bottom of each rectangular box represent the 75th and 25th percentiles respectively, with the median indicated with a solid line inside the box. Horizontal bars extending from each box represent more extreme values defined as 1.5-times the interquartile range (25th percentile subtracted from the 75th percentile) above the 75th percentile or below 25th percentile. Student's t-test was used to ascertain statistical significance. b) Bar plots representing the percentage of PCa patients that present genetic alterations in each DNA repair gene based on whether they have amplifications in *YWHAZ* (red bars), high or low *YWHAZ* mRNA levels (blue bars) and high or low 14-3-3ζ/δ levels (green bars). c) Comparative table of the percentage of patients that present genetic alterations in each DNA repair gene based on whether they have amplifications in *YWHAZ,* and high or low *YWHAZ* mRNA levels. Fisher's exact test was used to test the statistical significance of contingency tables of genetic alterations. Statistical significance was set at P≤0.05. * P≤0.05, ** P≤ 0.01, *** P<0.001.
