# Supplementary material for: The expression of YWHAZ and NDRG1 predicts aggressive outcome in human prostate cancer
Source: Commun Biol. 2021 Jan 22;4:103. doi: 10.1038/s42003-020-01645-2 (PMC7822895; doi:10.1038/s42003-020-01645-2)
Supplement: Supplementary file 2 — Description of Additional Supplementary Files [file 42003_2020_1645_MOESM2_ESM.pdf]

Description of Additional Supplementary Files

File Name: Supplementary Data 1
Description: List of PCa enriched proteins identified by LC ESI-MS/MS analysis. The table includes the protein and gene name, the theoretical molecular mass (KDa), the average number of PSMs, the average number of unique peptides, and the % coverage. PSMs = Peptide Spectrum Matches.

File Name: Supplementary Data 2
Description: List of BPH enriched proteins identified by LC ESI-MS/MS analysis. The table includes the protein and gene name, the theoretical molecular mass (KDa), the average number of PSMs, the average number of unique peptides, and the % coverage. PSMs = Peptide Spectrum Matches.

File Name: Supplementary Data 3
Description: Data sources for all of the publicly available Prostate Cancer datasets used within this work.
