# Supplementary material for: The expression of YWHAZ and NDRG1 predicts aggressive outcome in human prostate cancer
Source: Commun Biol. 2021 Jan 22;4:103. doi: 10.1038/s42003-020-01645-2 (PMC7822895; doi:10.1038/s42003-020-01645-2)
Supplement: Supplementary file 6 — Reporting Summary [file 42003_2020_1645_MOESM6_ESM.pdf]

nature research

Corresponding author(s): Geraldine Gueron

Last updated by author(s): Nov 18, 2020

Reporting Summary

Nature Research wishes to improve the reproducibility of the work that we publish. This form provides structure for consistency and transparency in reporting. For further information on Nature Research policies, see our Editorial Policies and the Editorial Policy Checklist.

Statistics

For all statistical analyses, confirm that the following items are present in the figure legend, table legend, main text, or Methods section.

n/a	Confirmed	
☐	☒	The exact sample size (*n*) for each experimental group/condition, given as a discrete number and unit of measurement
☐	☒	A statement on whether measurements were taken from distinct samples or whether the same sample was measured repeatedly
☐	☒	The statistical test(s) used AND whether they are one- or two-sided<br>*Only common tests should be described solely by name; describe more complex techniques in the Methods section.*
☐	☒	A description of all covariates tested
☐	☒	A description of any assumptions or corrections, such as tests of normality and adjustment for multiple comparisons
☐	☒	A full description of the statistical parameters including central tendency (e.g. means) or other basic estimates (e.g. regression coefficient) AND variation (e.g. standard deviation) or associated estimates of uncertainty (e.g. confidence intervals)
☐	☒	For null hypothesis testing, the test statistic (e.g. *F*, *t*, *r*) with confidence intervals, effect sizes, degrees of freedom and *P* value noted<br>*Give P values as exact values whenever suitable.*
☐	☒	For Bayesian analysis, information on the choice of priors and Markov chain Monte Carlo settings
☒	☐	For hierarchical and complex designs, identification of the appropriate level for tests and full reporting of outcomes
☒	☐	Estimates of effect sizes (e.g. Cohen's *d*, Pearson's *r*), indicating how they were calculated

Our web collection on statistics for biologists contains articles on many of the points above.

Software and code

Policy information about availability of computer code

Data collection	No software was used.
Data analysis	No software was used.

For manuscripts utilizing custom algorithms or software that are central to the research but not yet described in published literature, software must be made available to editors and reviewers. We strongly encourage code deposition in a community repository (e.g. GitHub). See the Nature Research guidelines for submitting code & software for further information.

Data

Policy information about availability of data

All manuscripts must include a data availability statement. This statement should provide the following information, where applicable:

- Accession codes, unique identifiers, or web links for publicly available datasets
- A list of figures that have associated raw data
- A description of any restrictions on data availability

The datasets generated during the current study are available in the ProteomeXchange (http://www.proteomexchange.org) repository, via with identifier PXD014291. Reviewer account details: Username: reviewer51767@ebi.ac.uk; Password: YsOSgbqT

Field-specific reporting

Life sciences study design

All studies must disclose on these points even when the disclosure is negative.

Sample size	Archival formalin-fixed and paraffin-embedded (FFPE) section tissues from 20 patients (ten radical prostatectomy specimens of treatment naïve PCa patients and ten BPH patients) were analyzed.
Data exclusions	LC ESI-MS/MS samples with less than 250 peptides were excluded from the subsequent analysis. The threshold was pre-established in order to exclude samples with low yield.
Replication	Three FFPE sections per patient were used for protein extraction.
Randomization	Patients were separated in two groups: 1) radical prostatectomy specimens of treatment naïve PCa patients and 2) BPH patients.
Blinding	Blinding was not relevant to this study. Patients were separated in two groups: 1) radical prostatectomy specimens of treatment naïve PCa patients and 2) BPH patients.

Reporting for specific materials, systems and methods

We require information from authors about some types of materials, experimental systems and methods used in many studies. Here, indicate whether each material, system or method listed is relevant to your study. If you are not sure if a list item applies to your research, read the appropriate section before selecting a response.

Materials & experimental systems

n/a	Involved in the study	
☒	☐	Antibodies
☒	☐	Eukaryotic cell lines
☒	☐	Palaeontology and archaeology
☒	☐	Animals and other organisms
☐	☒	Human research participants
☒	☐	Clinical data
☒	☐	Dual use research of concern

Methods

n/a	Involved in the study	
☒	☐	ChIP-seq
☒	☐	Flow cytometry
☒	☐	MRI-based neuroimaging

Human research participants

Policy information about studies involving human research participants

Population characteristics	Archival formalin-fixed and paraffin-embedded (FFPE) section tissues from 20 patients (ten radical prostatectomy specimens of treatment naïve PCa patients and ten BPH patients) were analyzed. PCa patients age is between 54-92, tumor stages are variable between patiens, and patient Gleason scores are between 5-8.
Recruitment	No recruitment was done. Archival FFPE sections were used. BRISQ reporting guidelines and Tier 1 characteristics are provided.
Ethics oversight	Written informed consent and institutional review board approval from the teaching hospital "Hospital de Clínicas José de San Martín" in Buenos Aires, Argentina, were acquired.

Note that full information on the approval of the study protocol must also be provided in the manuscript.
